# Epithelial and Stromal Cells of Bovine Endometrium Have Roles in Innate Immunity and Initiate Inflammatory Responses to Bacterial Lipopeptides In Vitro via Toll-Like Receptors TLR2, TLR1, and TLR6

**DOI:** 10.1210/en.2013-1822

**Published:** 2014-01-17

**Authors:** Matthew L. Turner, James G. Cronin, Gareth D. Healey, Iain Martin Sheldon

**Affiliations:** Institute of Life Science, School of Medicine, Swansea University, Singleton Park, Swansea SA2 8PP, United Kingdom

## Abstract

Bacteria often infect the endometrium of cattle to cause endometritis, uterine disease, and infertility. Lipopeptides are commonly found among bacteria and are detected by the Toll-like receptor (TLR) cell surface receptor TLR2 on immune cells. Heterodimers of TLR2 with TLR1 or TLR6 activate MAPK and nuclear factor-κB intracellular signaling pathways to stimulate inflammatory responses. In the endometrium, epithelial and stromal cells are the first to encounter invading bacteria, so the present study explored whether endometrial cells can also mount inflammatory responses to bacterial lipopeptides via TLRs. The supernatants of pure populations of primary bovine endometrial epithelial and stromal cells accumulated the cytokine IL-6 and the chemokine IL-8 in response to triacylated or diacylated bacterial lipopeptides. The accumulation of IL-6 and IL-8 in response to triacylated lipopeptides was reduced by small interfering RNA targeting TLR2 or TLR1 but not TLR6, whereas cellular responses to diacylated lipopeptide were reduced by small interfering RNA targeting TLR2, TLR1, or TLR6. Both lipopeptides induced rapid phosphorylation of ERK1/2, p38, and nuclear factor-κB in endometrial cells, and inhibitors of ERK1/2 or p38 limited the accumulation of IL-6. The ovarian steroids estradiol and progesterone had little impact on inflammatory responses to lipopeptides. The endometrial epithelial and stromal cell responses to lipopeptides via TLR2, TLR1, and TLR6 provide a mechanism linking a wide range of bacterial infections to inflammation of the endometrium.

The endometrium lining the uterus of mammals is often infected by Gram-negative and Gram-positive bacteria ascending through the cervix, around the time of coitus or parturition (1, 2). Postpartum bacterial infections of the uterus are particularly common causes of disease in dairy cows, whereas sexually transmitted infections often cause infertility in beef breeds of *Bos taurus* and *Bos indicus* (1). Postpartum uterine disease is important because it affects ∼40% of dairy cows, compromising animal welfare, causing infertility, and diminishing the production of food for human consumption. The combined cost for treatment of postpartum uterine disease, lost milk production, and replacement of infertile animals is about $2 billion per annum for the combined North American and European Union dairy industries (2). *Escherichia coli* is the most common Gram-negative pathogen isolated from the postpartum uterus of cattle, and well-established mechanisms link endometritis to the action of the lipopolysaccharide (LPS) cell wall component of Gram-negative bacteria (1, 3). The Gram-positive bacteria *Trueperella pyogenes*, *Fusobacterium necrophorum*, *Bacteroides*, and *Prevotella* species are also often isolated from the diseased endometrium postpartum (4). Sexually transmitted infections commonly involve *Trichomonas fetus*, *Mycoplasma* species, and *Campylobacter fetus*. However, apart from Gram-negative bacteria, it is not clear how bacteria are detected in the endometrium or whether microbial factors other than LPS stimulate endometrial cellular responses.

Innate immunity is an ancient system of cellular defense based on pattern recognition receptors that bind pathogen-associated molecular patterns (PAMPs), which are found in prokaryotes but not eukaryotes (5–7). The first functional mammalian pattern recognition receptor to be identified was Toll-like receptor (TLR) 4 on murine macrophages, which binds LPS (8). Gram-positive bacteria do not posses LPS but all bacteria, including Gram-positive bacteria and *Mycoplasma*, express lipopeptides (9). Triacylated lipopeptides are the most common in Gram-negative bacteria and bind TLR2, which heterodimerizes with TLR1 in mice, whereas diacylated lipopeptides are found in Gram-positive bacteria or *Mycoplasma* and bind TLR2/TLR6 heterodimers (10, 11). Synthetic triacylated and diacylated lipopeptides are used to examine TLR2 function because preparations of native bacterial lipopeptides are often contaminated with ligands for other TLRs, such as LPS (12–14). In murine or human hematopoietic cells, engagement of TLR2/TLR1 and TLR2/TLR6 heterodimers by triacylated and diacylated lipopeptides, respectively, activates MAPK and nuclear factor of κ light polypeptide gene enhancer in B-cells (NFκB) intracellular signaling pathways. Phosphorylation of p38 (also known as MAPK14) or ERK1/2 (also known as MAPK3/1), or phosphorylation of p65 NFκB leads to gene transcription and secretion of cytokines such as IL-1β, IL-6, and TNFα, and chemokines such as IL-8 (5, 6).

In cattle, mRNA transcripts for cytokines *IL1B* and *IL6*, and the chemokine *IL8* are more abundant in the endometrium of diseased than normal animals (15, 16). Epithelial and stromal cells are the first to encounter bacteria invading the endometrium, and these cells express *TLR4* mRNA and secrete IL-6 and IL-8 in response to LPS, via TLR4-dependent activation of p38, ERK1/2, and NFκB signaling pathways (17, 18). However, whereas endometrial cells express *TLR2, TLR1*, and *TLR6* mRNA, there is little evidence in any species about whether primary endometrial cells detect bacterial lipopeptides (2, 19). The genomic conservation for each of the *TLR2*, *TLR1*, and *TLR6* genes is >96% among *Bos taurus* and *Bos indicus*, but conservation between *B. taurus* and *Homo sapiens* is 84% to 88% and between *B. taurus* and *Mus musculus* is only 75% to 78%. There are also species-dependent variations in responses associated with innate immunity (20). Thus, to explore whether TLR2 is important in the bovine endometrium, it is essential to examine tissues from the target species.

The present study tested the hypothesis that bovine endometrial epithelial and stromal cells mount inflammatory responses to bacterial lipopeptides via TLR2, TLR1, and TLR6 pathways. Bovine endometrial cells produced IL-6 and IL-8 in response to triacylated lipopeptide, and the response was blunted using small interfering RNA (siRNA) targeting TLR2 or TLR1. There were similar cellular responses to diacylated lipopeptide, and siRNA targeting TLR2, TLR1, or TLR6 reduced the accumulation of IL-6 and IL-8. Phosphorylation of p38, ERK1/2, and NFκB in response to lipopeptides provided further evidence of TLR2 function. Furthermore, cellular responses to lipoproteins were reduced by inhibitors targeting p38 or ERK1/2. In summary, endometrial cells have roles in innate immunity to sense and respond to triacylated and diacylated lipopeptides, which provides a mechanism linking endometritis to a wide range of bacteria.

## Materials and Methods

### Isolation and culture of endometrial cells

Uteri with no gross evidence of genital disease or microbial infection and peripheral blood samples were collected from 2.2 ± 0.1-year-old postpubertal mixed-breed beef cattle within 15 minutes of slaughter at a local slaughterhouse; the studies used >80 animals. Postpartum cattle were not used because experiments would be confounded by the usual ubiquitous bacterial contamination of the uterus and disruption of the epithelium typical of the puerperal endometrium (2, 15, 16, 21). The stage of the reproductive cycle was determined by examination of ovarian morphology and vasculature, as described previously (22); uteri at ovarian stage I (days 1–4 of the estrous cycle) were selected for endometrial cell culture because peripheral plasma progesterone concentrations are basal, similar to those of postpartum cows. The uteri were kept on ice for ∼1 hour until further processing at the laboratory.

Endometrial tissue was dissected and processed as described previously (17, 18). In brief, tissue was digested in 25 mL of sterile digestive solution, made by dissolving 50 mg of trypsin (Sigma-Aldrich), 50 mg of collagenase II (Sigma-Aldrich), 100 mg of BSA (Sigma-Aldrich), and 10 mg of DNase I (Sigma-Aldrich) in 100 mL of Hanks' balanced salt solution (Sigma). After a 1-hour incubation in a shaking water bath at 37°C, the cell suspension was filtered through a 40-μm mesh (Fisher Scientific) to remove undigested material. The filtrate was resuspended in washing medium, composed of Hanks' balanced salt solution with 10% heat-inactivated fetal bovine serum (FBS) (Biosera). The suspension was centrifuged at 700 × *g* for 7 minutes, and after 2 further washes in washing medium, the cells were resuspended in endometrial cell culture medium (RPMI 1640 medium, 10% FBS, 50 IU/mL of penicillin, 50 μg/mL of streptomycin, and 2.5 μg/mL of amphotericin B; all from Sigma-Aldrich). All experiments used the same batch of FBS, which during previous batch testing yielded the lowest concentrations of cytokines and chemokines in supernatants of endometrial cells and macrophages cultured in endometrial cell culture medium (10 batches from 4 companies tested). The cells were cultured in 75-cm^2^ flasks (Greiner Bio-One) for 18 hours to allow selective attachment of stromal cells, with the remaining epithelial cell suspension transferred to a new flask. The cells were incubated at 37°C in a humidified atmosphere of air with 5% CO_2_, and endometrial cell culture medium was changed every 48 hours. Epithelial and stromal cell populations were distinguished by cell morphology, and the absence of immune cell contamination was confirmed by the absence of CD45, as described previously (17, 23). The epithelial or stromal cells were resuspended and plated at 1.5 × 10^5^ cells/mL in 1 mL for 24-well plates (TPP) to examine cytokine or chemokine responses, in 2 mL for 12-well plates for collection of cells for immunoblotting, and in 6-well plates to test the role of TLRs using siRNA, as described below.

To examine endometrial cell purity, endometrial epithelial cells, stromal cells, and whole blood samples were suspended in BSA stain buffer (BD Biosciences) at 1 × 10^7^ cells/mL for fluorescence-activated cell sorting (FACS) analysis. For surface staining, RPE-conjugated mouse anti-CD45 (MA1–81458; Thermo Scientific) hematopoietic cell marker or the isotype control, RPE-conjugated anti-mouse IgG1 (PA5–33180; Thermo Scientific) was added to 100-μL aliquots of the purified cells and incubated on ice for 30 minutes in the dark. After staining, cells were washed twice by centrifuging at 520 × *g* for 7 minutes, removing the supernatant, and resuspending in BSA stain buffer. For intracellular staining, 100-μL aliquots of the purified cell populations, suspended at 1 × 10^7^ cells/mL in BSA stain buffer, were fixed with BD Cytofix (BD Biosciences) for 30 minutes on ice, washed twice as above, and then permeabilized with Perm Buffer III (BD Biosciences) for a further 30 minutes on ice. After permeabilization, cells were washed twice and stained with allophycocyanin-conjugated mouse anti–pan-cytokeratin (ab106166; Abcam) as a marker of epithelia, fluorescein isothiocyanate–conjugated mouse anti-vimentin (BM5501F; Acris Antibodies) as a mesenchymal cell marker, or the isotype controls, allophycocyanin-conjugated anti-mouse IgG1 (406609; BioLegend Inc) or fluorescein isothiocyanate–conjugated anti-mouse IgG2a (407105; BioLegend Inc), respectively, for 30 minutes on ice, in the dark. Cells were then washed twice, as above, and resuspended in BSA stain buffer for analysis. Analysis was performed on a BD FACSAria III cell sorter instrument using BD FACSDiva v6.1 software (BD Biosciences). At least 1 × 10^4^ cells were acquired per sample, and dead cells were excluded by selective scatter gating.

### Endometrial cell responses to PAMPs

To evaluate responses to PAMPs, endometrial cells were treated with ultrapure preparations of the triacylated lipopeptide *N*-palmitoyl-*S*-[2,3-bis(palmitoyloxy)-(2*RS*)-propyl]-[*R*]-cysteinyl-[*S*]-seryl-[*S*]-lysyl-[*S*]-lysyl-[*S*]-lysyl-[*S*]-lysine (PAM, Pam3CSK4), diacylated lipopeptide [*S*-(2,3-bispalmitoyloxypropyl) Cys-Gly-Asp-Pro-Lys-His-Pro-Lys-Ser-Phe (FSL-1, Pam2CGDPKHPKSF), and LPS from *E. coli* 0111:B4 as a positive control (all from InvivoGen). Epithelial or stromal cells 80% confluent in 24-well culture plates were treated with endometrial cell culture medium containing vehicle or 10-fold increasing concentrations from 0.1 to 1000 ng/mL PAM, FSL-1, or LPS. These concentrations span the range recommended by the manufacturer and reflect concentrations of LPS measured in diseased cattle (14 days postpartum: uterine fluid, 18–262 μg/mL LPS; and peripheral plasma, 0–0.6 ng/mL LPS) (24). After 24 hours, cell-free culture supernatants were collected and stored at −20°C for analysis of inflammatory mediators by ELISA. For each PAMP, experiments were repeated on 3 independent occasions, with treatments applied to duplicate replicate wells.

To evaluate the role of TLRs in cellular responses to lipopeptides, siRNA was used to target *TLR2*, *TLR1*, and *TLR6*. The siRNAs were designed using siDESIGN Center (www.thermoscientificbio.com/design-center/) and duplex sequences were as follows: *TLR2*, sense GGACAGAAUUAGACACCUAUU and antisense UAGGUGUCUAAUUCUGUCCUU (Genbank accession number NM_174197.2); *TLR1*, sense UGAUGAAAGUGAAGGAAAUUU and antisense AUUUCCUUCACUUUCAUCAUU (NM_001046504.1); and *TLR6*, sense CAGAAUAGUUUCACAGAUAUU and antisense UAUCUGUGAAACUAUUCUGUU (NM_001001159.1). The RNAiMAX-RNAi duplex complexes were formed by addition of 50 pmol of target siRNA or ON-TARGETplus nontargeting siRNA no. 1 (scramble; Dharmacon) to 500 μL of Opti-MEM I Reduced Serum Medium without antibiotics (Invitrogen) to each well of a 6-well culture plate, with 7.5 μL of Lipofectamine RNAiMAX (Invitrogen) added to the siRNA duplex and control wells and incubated for 20 minutes at room temperature. Growing 2 × 10^5^ epithelial cells or 1.5 × 10^5^ stromal cells were seeded into each well in 2.5 mL of complete RPMI 1640 growth media without antibiotics to yield approximately 50% confluence. After 24 hours, the cells were washed with Dulbecco PBS and lysed using Buffer RLT for analysis of TLR mRNA by quantitative PCR. Alternatively, cells were treated with control endometrial cell culture medium or medium containing 100 ng/mL PAM or FSL-1, because these concentrations stimulated cellular responses; after 24 hours, culture supernatants were collected and stored at −20°C for analysis of inflammatory mediators by ELISA, and cell survival was evaluated by the 3-(4,5-dimethylthiazol-2-yl)-2,5-diphenyltetrazolium bromide (MTT) assay (see below). The experiments were repeated on 3 independent occasions for each TLR.

To determine whether intracellular MAPK or NFκB signaling pathways were activated by lipopeptides, epithelial or stromal cells in 12-well culture plates were cultured in Opti-MEM I Reduced Serum Medium or medium containing 100 ng/mL PAM or FSL-1. Cells were collected after 0, 5, 10, 15, 20, and 25 minutes of treatment, washed with Dulbecco PBS, lysed by addition of 200 μL of Phosphosafe Extraction Reagent (Merck), and stored at −80°C for analysis by immunoblotting. The experiments were repeated on 3 independent occasions.

To further evaluate the role of intracellular signaling pathways in the response to lipopeptides, inhibitors targeting MAPK were tested using previously validated concentrations for bovine cells, although inhibitors of NFκB were not used because they cause cell death (18). Epithelial or stromal cells cultured in 24-well plates were treated with an inhibitor of ERK1/2 activation (10 μM ERK activation inhibitor peptide I, catalog no. 328000; Merck Chemicals) or an inhibitor of p38 (10 μM InSolution, SB203580; Merck Chemicals) for 30 minutes before and during treatment with endometrial cell culture medium containing 100 ng/mL PAM or FSL-1; the controls for each experiment were an equivalent volume of medium and vehicle, and an equivalent volume of medium and each inhibitor. Inhibitors were selected on the basis of evidence of selectivity, suppression of endometrial cell inflammatory responses to LPS, and preliminary experiments showing reduced phosphorylation of MAPK (Supplemental Figure 1 published on The Endocrine Society's Journals Online web site at http://end.endojournals.org) (18, 25, 26). After 6 hours, cell supernatants were collected and stored at −20°C for analysis of IL-6 by ELISA, and cell survival was evaluated by the MTT assay. The experiments were repeated on 3 independent occasions, with treatments applied to duplicate replicate wells.

To evaluate the impact of ovarian steroids on cellular responses to lipopeptides, endometrial cells were treated with concentrations of estradiol or progesterone that reflect the concentration in peripheral plasma during estrus or the luteal phase, respectively. Epithelial or stromal cells in 24-well plates were cultured in RPMI 1640 without phenol red (Sigma-Aldrich) containing 10% charcoal-stripped FBS, with 50 IU/mL of penicillin, 50 μg/mL of streptomycin, and 2.5 μg/mL amphotericin B and treated with 3 pg/mL estradiol (Sigma-Aldrich), 5 ng/mL progesterone (Sigma-Aldrich), a combination of estradiol and progesterone, or 5 ng/mL dexamethasone (Sigma-Aldrich) as a positive control. After 24 hours, the cells were treated with vehicle or 100 ng/mL PAM or FSL-1 for a further 24 hours, and then the cell supernatants were collected and stored at −20°C for analysis of IL-6 by ELISA.

### ELISA

Concentrations of IL-1β, IL-6, IL-8, and TNFα in cell culture supernatants were measured by ELISA according to the manufacturer's instructions (Bovine IL-1β Screening Set ESS0027 and Bovine IL-6 Screening Set ESS0029 [Thermo Scientific] and Bovine TNFα DuoSet DY2279 and Human CXCL8/IL-8 DuoSet DY208 [R&D Systems Europe Ltd]). The human IL-8 assay has previously been shown to cross-react with bovine IL-8 (27). The inter-assay and intra-assay coefficients of variation were all <10%; the limits of detection were 12.5 pg/mL for IL-1β, 75.0 pg/mL for IL-6, 83.2 pg/mL for TNFα, and 5.7 pg/mL for IL-8.

### Immunoblotting

Proteins were quantified using the DC Assay (Bio-Rad) and separated (10 μg/lane) using 12% (vol/vol) SDS-PAGE with prestained molecular weight markers (Bio-Rad) in parallel lanes. After electrophoresis, proteins were transferred to a polyvinylidene difluoride membrane (Bio-Rad); nonspecific sites were blocked using a solution of 5% (wt/vol) BSA (Sigma-Aldrich) in Tris-buffered saline and 0.1% Tween 20 (TBS/T) overnight at 4°C with gentle agitation. Membranes were probed with antibodies targeting total and phosphorylated forms of ERK1/2 (anti-ERK1/2 [AB17942; [Abcam] and anti-MAPK activated diphosphorylated ERK1/2 [M8159; Sigma-Aldrich]), p38 (MAPK p38/MAPK14 [AP03041SU-N; Acris antibodies, 2B Scientific] and MAPK p38/MAPK14pThr180/pTyr182 [AP05898PU-N, Acris antibodies, 2B Scientific]), and NFκB (NFκB p65 [4764S; New England Biolabs] and phospho NFκB p65(Ser536) [3033L; New England Biolabs]), with protein loading confirmed using antibodies against α-tubulin (bovine α-tubulin, A11126; Invitrogen) or β-actin (bovine anti-β actin, ab8226; Abcam); the antibodies are further detailed in Supplemental Table 1 and were selected on the basis of recognition of immunoreactive proteins of appropriate molecular weight and previous publications (18, 28, 29). Membranes were incubated with primary antibodies diluted in 5% (wt/vol) BSA in TBS/T for 2 hours with gentle agitation. After incubation, membranes were washed 4 times for 5 minutes in TBS/T. Membranes were then incubated with secondary horseradish peroxidase–conjugated antibody in 5% (wt/vol) BSA in TBS/T for 1.5 hours and washed 4 times for 5 minutes in TBS/T. Immunoreactive proteins were visualized using enhanced chemiluminescence (Western C; Bio-Rad). The average peak densities of unsaturated bands were analyzed using Quantity One software (Bio-Rad) with the band intensities for each phosphorylated protein normalized against the bands for their respective total protein, and data are presented relative to cells at time 0.

### MTT assay

The number of cells per well at the end of treatment was evaluated by MTT assays. After removal of cell culture supernatants, cells were incubated in 250 μL of 0.5 mg/mL MTT (Sigma-Aldrich) for 1 hour at 37°C, with 5% CO_2_ in air, in a humidified incubator. Then the medium was removed, and the cells were washed with Dulbecco PBS before lysis using dimethylsulfoxide, with OD measured at 570 nm using a microplate reader (POLARstar Omega; BMG Labtech). In addition, in preliminary studies, the number of cells per well at the start of treatment did not differ significantly (all within ±9%).

### Quantitative PCR

At the end of each experiment, endometrial cells were washed with 1 mL of PBS, and RNA was extracted using an RNeasy Mini Kit and automated QIAcube system (QIAGEN), according to the manufacturer's instructions. Extracted RNA was quantified using a NanoDrop ND1000 spectrophotometer (Labtech), and the purity of each sample was determined by the ratio A_260_/A_280_. An A_260_/A_280_ ratio of between 1.8 and 2.1 was considered suitable for further investigation. For cDNA synthesis, 1 μg of total RNA was added to a genomic DNA elimination reaction, followed by reverse transcription to cDNA (QuantiTect Reverse Transcription Kit; QIAGEN), according to the manufacturer's instructions. Quantitative PCR was performed according to previously published guidelines (30) using QuantiFast SYBR Green (QIAGEN) and the iQ5 RT-PCR detection system (Bio-Rad) with software version 2.1.97.1001. Each sample was assayed in triplicate using intron-spanning primers designed with the online software NCBI/Primer-Basic Local Alignment Search Tool (National Center for Biotechnology Information). Target-specific primers generated PCR products of up to 200 bp (Supplemental Table 2). The *ACTB* gene, which was invariant across the treatments used, was chosen as the reference gene for normalization, and the relative quantification method was used to quantify target gene mRNA within samples (31). To generate standard curves, total RNA extracted from stromal cells that had been treated with 100 ng/mL LPS for 24 hours was reverse transcribed to cDNA, as described previously. Ten-fold serial dilutions of this reference cDNA were prepared (undiluted to 1 × 10^−3^) in RNase-free water. PCR conditions consisted of 25-μL reactions containing 2 μL of cDNA, and thermal cycling parameters were 1 cycle of 95°C for 5 minutes, followed by 40 cycles of 95°C for 30 seconds and 60°C for 60 seconds; a melt curve was subsequently generated to confirm the specificity and identity of PCR products. For each sample, target and reference gene mRNA abundance was determined from the appropriate standard curve (quantification cycle, C_q_). Changes in mRNA abundance between samples were then determined from the ratio of the target gene C_q_ to the reference gene C_q_.

### Statistical analysis

Data are presented as the arithmetic means and SEM. Statistical analyses were performed using SPSS 16.0 (SPSS Inc) with the animal as the designated statistical unit, and a value of *P* < .05 was considered statistically significant. Treatments were compared using ANOVA with the Dunnett post hoc test for normally distributed data or by the Mann-Whitney test for nonparametric data. Because we were aware of between-animal biological variation for primary cell cultures, for analysis of the impact of siRNA or inhibitors, the concentrations of inflammatory mediators were expressed for each animal as a percentage of the response to treatment with each PAMP.

## Results

### Endometrial cells respond to PAMPs

**Figure 1.**
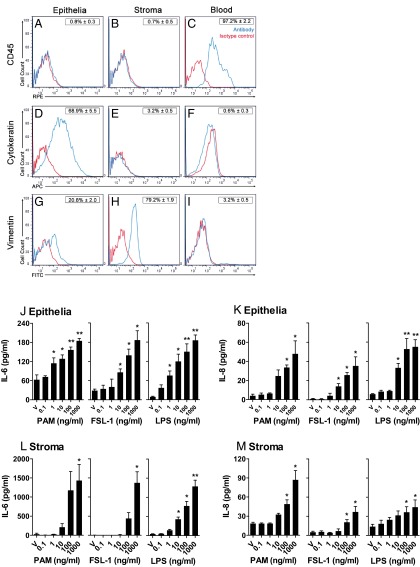
Endometrial cell purity and response to PAMPs. Purified populations of epithelial or stromal cells or whole blood samples were stained for the cell surface hematopoietic cell marker CD45 or the intracellular epithelial and mesenchymal markers cytokeratin and vimentin, respectively. Flow cytometry histograms present data in blue for cells stained with CD45 (A, B, and C), cytokeratin (D, E, and F), or vimentin (G, H, and I), and in red for the cognate isotype control. Histograms depict a representative sample from 1 animal using 10,000 cells for each analysis. Values in boxes are the mean percent staining compared with that for the relevant isotype control ± SEM, for 3 independent animals. Epithelial (J and K) and stromal cells (L and M) isolated from bovine endometrium were cultured for 24 hours in medium containing vehicle (V) or 0.1, 1, 10, 100, to 1000 ng/mL PAM, FSL-1, or LPS. Supernatants were harvested to measure the accumulation of IL-6 (J and L) and IL-8 (K and M) by ELISA. Data are presented as mean + SEM and represent 3 independent experiments for each PAMP. Values differ significantly from those for vehicle when data were analyzed by ANOVA using the Dunnett pairwise multiple comparison *t* test: *, *P* < .05; **, *P* < .01.

Endometrial cells were not contaminated with hematopoietic cells as determined by FACS analysis for CD45 expression (Figure 1, A–C). Epithelial but few stromal cells expressed the epithelial marker cytokeratin (Figure 1, D–F), and more stromal than epithelial cells expressed the mesenchymal cell marker vimentin (Figure 1, G–I).

The supernatants of endometrial epithelial or stromal cells accumulated more IL-6 and IL-8 in response to 24 hours of treatment with PAM or FSL-1 compared with those for the vehicle control (Figure 1, J–M). The concentration-dependent accumulation of IL-6 and IL-8 after treatment with PAM or FSL-1 was similar in magnitude to that of cells treated with the prototypical PAMP, LPS. However, the concentrations of IL-1β and TNFα were below the limits of detection of the assays for PAM, FSL-1, and LPS. Cell survival and variations in cell density between culture wells were evaluated by the MTT assay, which was not significantly affected by treatment with PAM (97.1 ± 4.2% [epithelium] and 105.5 ± 3.0% [stroma] of vehicle), FSL-1 (106.1 ± 10.9% [epithelium] and 115.2 ± 3.8% [stroma] of vehicle), or LPS (111.4 ± 10.9% [epithelium] and 125.6 ± 12.2% [stroma] of vehicle). Because IL-6 and IL-8 responses were detectable at concentrations of 100 ng/mL PAM or 100 ng/mL FSL-1, these concentrations of lipopeptides were used for subsequent experiments.

### Endometrial cells respond to lipopeptides via TLRs

**Figure 2.**
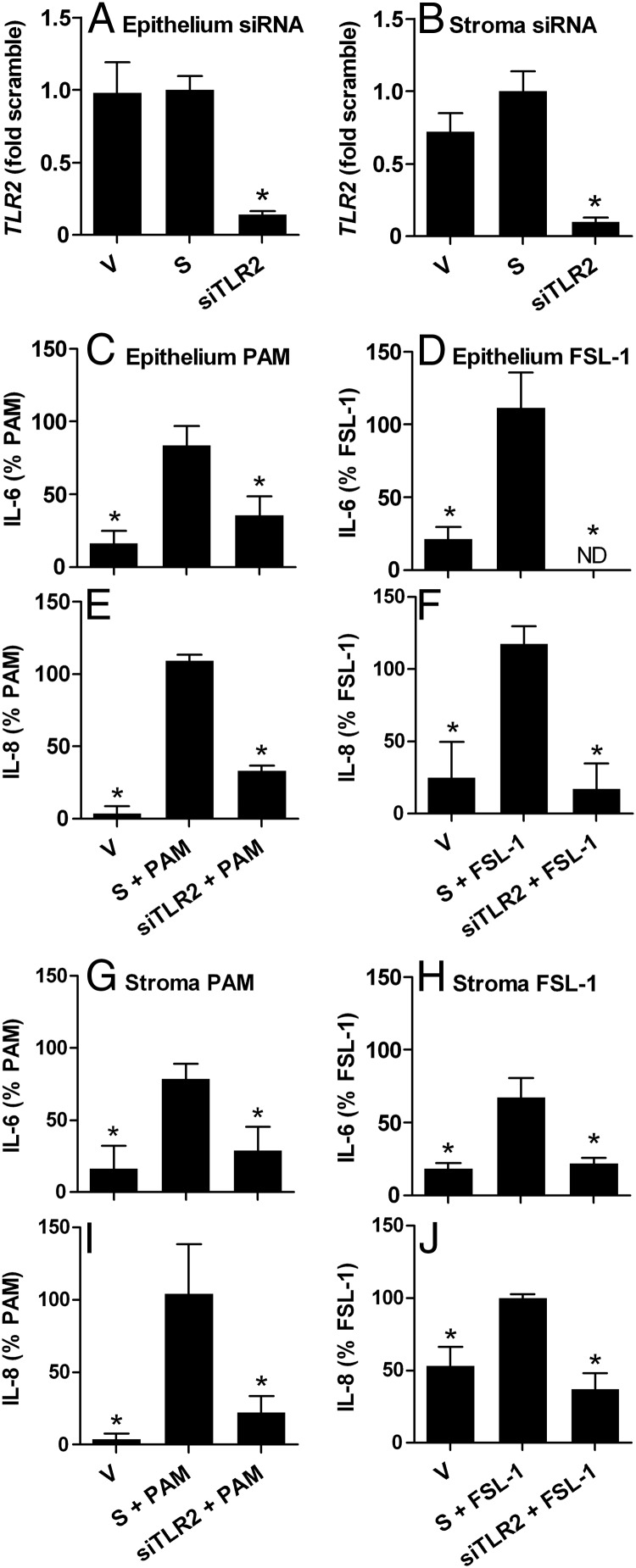
Attenuation of endometrial cell responses to lipopeptide PAMPs by siRNA targeting *TLR2*. Epithelial (A and C–F) and stromal cells (B and G–J) isolated from bovine endometrium were treated with vehicle (V), nontargeting scramble siRNA (S), or siRNA targeting *TLR2* (siTLR2) before treatment with control medium or medium containing 100 ng/mL PAM or 100 ng/mL FSL-1. A and B, Cells in control medium were collected, and the expression of *TLR2* mRNA was quantified by PCR relative to *ACTB* expression. Data are presented as mean + SEM fold of mRNA expression in cells treated with scramble siRNA from 3 independent experiments. Values differ from those for scramble and vehicle when analyzed by the Mann-Whitney *U* test: *, *P* < .05. C–J, Culture supernatants were harvested to measure the accumulation of IL-6 and IL-8 by ELISA, and results are expressed as the percentage of treatment with each PAMP from independent experiments (PAM, n = 4; FSL-1, n = 3). Data are presented as mean + SEM, and values differ significantly from those for scramble siRNA when analyzed by ANOVA using the Dunnett pairwise multiple comparison *t* test: *, *P* < .05.

**Figure 3.**
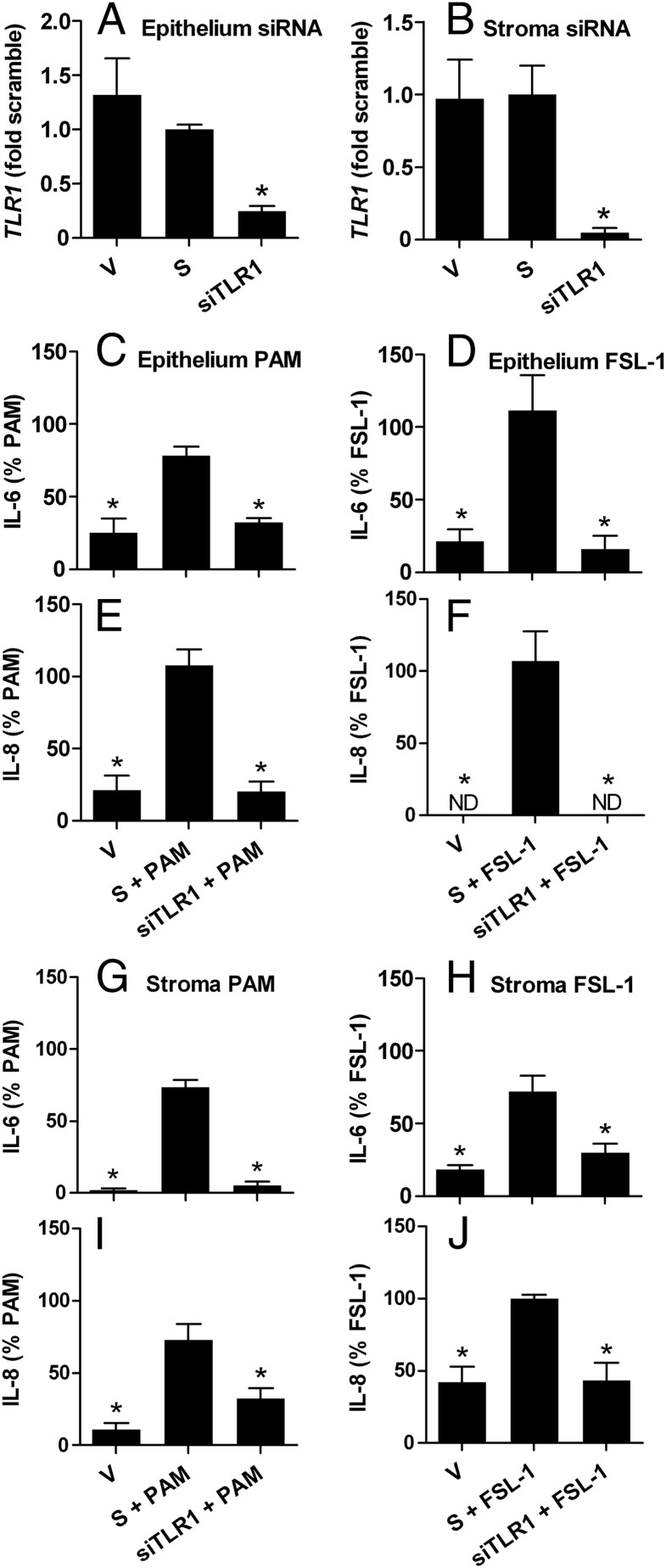
Attenuation of endometrial cell responses to lipopeptide PAMPs by siRNA targeting *TLR1*. Epithelial (A and C–F) and stromal cells (B and G–J) isolated from bovine endometrium were treated with vehicle (V), nontargeting scramble siRNA (S), or siRNA targeting *TLR1* (siTLR1) before treatment with control medium or medium containing 100 ng/mL PAM or 100 ng/mL FSL-1. A and B, Cells in control medium were collected, and the expression of *TLR1* mRNA was quantified by PCR relative to *ACTB* expression. Data are presented as mean ± SEM fold of mRNA expression in cells treated with scramble siRNA from 3 independent experiments. Values differ from those for scramble and vehicle when analyzed by the Mann-Whitney *U* test: *, *P* < .05. C–J, Culture supernatants were harvested to measure the accumulation of IL-6 and IL-8 by ELISA, and results are expressed as the percentage of treatment with each PAMP from independent experiments (PAM, n = 4; FSL-1, n = 4). Data are presented as mean + SEM, and values differ significantly from those for scramble siRNA, when analyzed by ANOVA using the Dunnett pairwise multiple comparison *t* test: *, *P* < .05.

**Figure 4.**
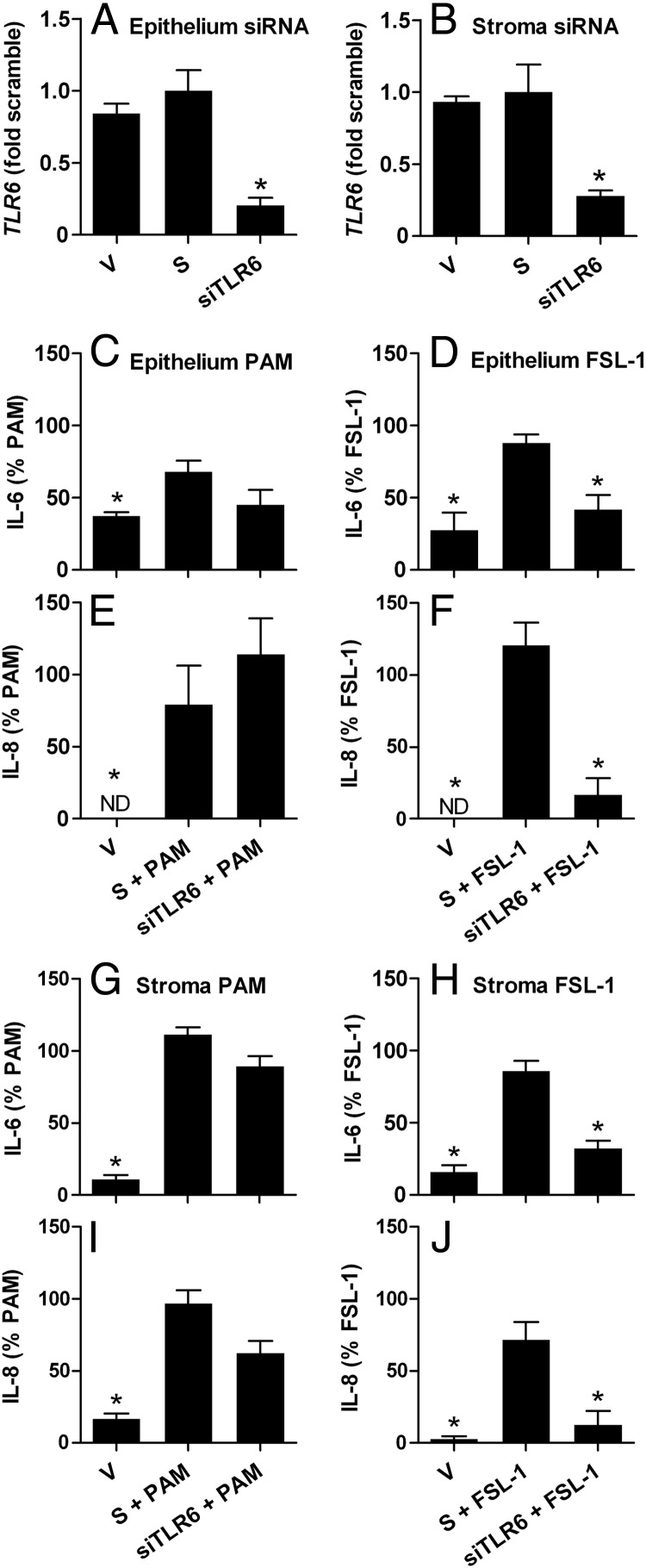
Attenuation of endometrial cell responses to lipopeptide PAMPs by siRNA targeting *TLR6*. Epithelial (A and C–F) and stromal cells (B and G–J) isolated from bovine endometrium were treated with vehicle (V), nontargeting scramble siRNA (S), or siRNA targeting *TLR6* (siTLR6) before treatment with control medium or medium containing 100 ng/mL PAM or 100 ng/mL FSL-1. A and B, Cells in control medium were collected, and the expression of *TLR6* mRNA was quantified by PCR relative to *ACTB* expression. Data are presented as means ± SEM fold of mRNA expression in cells treated with scramble siRNA from 3 independent experiments. Values differ from those for scramble and vehicle when analyzed by the Mann-Whitney *U* test: *, *P* < .05. C–J, Culture supernatants were harvested to measure the accumulation of IL-6 and IL-8 by ELISA, and results are expressed as the percentage of treatment with each PAMP from independent experiments (PAM, n = 4; FSL-1, n = 4). Data are presented as mean + SEM, and values differ significantly from those for scramble siRNA when analyzed by ANOVA using the Dunnett pairwise multiple comparison *t* test: *, *P* < .05; ND, below limits of detection.

To evaluate the role of TLRs in responses to lipopeptides, endometrial epithelial and stromal cells were transfected with siRNA targeting *TLR2, TLR1*, or *TLR6*, which reduced mRNA expression for *TLR2* (Figure 2, A and B), *TLR1* (Figure 3, A and B), and *TLR6* (Figure 4, A and B), compared with that for cells transfected with scramble siRNA. None of the siRNA significantly affected the basal accumulation of IL-6 or IL-8 from cells cultured in control medium (Supplemental Figure 2) or cell survival or variation in cell density between culture wells (MTT assay ODs all within ±14% of that for cells in control medium). The supernatants of epithelial cells treated with PAM or FSL-1 for 24 hours had increased accumulation of IL-6 (control, 100.1 ± 40.8 pg/mL; PAM, 190.6 ± 59.0 pg/mL; and FSL-1, 149.3 ± 48.1 pg/mL; n = 9, *P* < .05) and IL-8 (control, 0.4 ± 0.3 pg/mL; PAM, 10.3 ± 2.4 pg/mL; and FSL-1, 26.6 ± 3.5 pg/mL; n = 9, *P* < .05), and stromal cells treated with PAM or FSL-1 had increased accumulation of IL-6 (control, 27.8 ± 9.9 pg/mL; PAM, 1382.9 ± 380.0 pg/mL; and FSL-1, 148.6 ± 27.4 pg/mL; n = 9, *P* < .05) and IL-8 (control, 4.3 ± 2.5 pg/mL; PAM, 31.6 ± 11.3 pg/mL; and FSL-1, 20.2 ± 3.8 pg/mL; n = 9, *P* < .05). The concentrations of inflammatory mediators are expressed as a percentage of the response to PAM or FSL-1 to examine the impact of the targeting siRNA. The effect of siRNA targeting *TLR2* on endometrial cellular responses to lipopeptides was examined first because lipopeptides are bound by TLR2 irrespective of whether they are triacylated or diacylated (10). Indeed, siRNA targeting *TLR2* reduced the accumulation of IL-6 and IL-8 in the supernatants of epithelial cells treated with PAM (Figure 2, C and E) or FSL-1 (Figure 2, D and F) and in stromal cells treated with PAM (Figure 2, G and I) or FSL-1 (Figure 2, H and J), compared with cells transfected with scramble siRNA. The use of siRNA targeting *TLR1* also reduced the accumulation of IL-6 and IL-8 in the supernatants of epithelial cells treated with PAM (Figure 3, C and E) or FSL-1 (Figure 3, D and F) and stromal cells treated with PAM (Figure 3, G and I) or FSL-1 (Figure 3, H and J). However, siRNA targeting *TLR6* reduced the accumulation of IL-6 and IL-8 in the supernatants of epithelial cells treated with FSL-1 (Figure 4, D and F) but not with PAM (Figure 4, C and E). Similarly, siRNA targeting *TLR6* reduced the accumulation of IL-6 and IL-8 in the supernatants of stromal cells treated with FSL-1 (Figure 4, H and J) but not with PAM (Figure 4, G and I).

### Activation of MAPK and NFκB in response to lipopeptides

**Figure 5.**
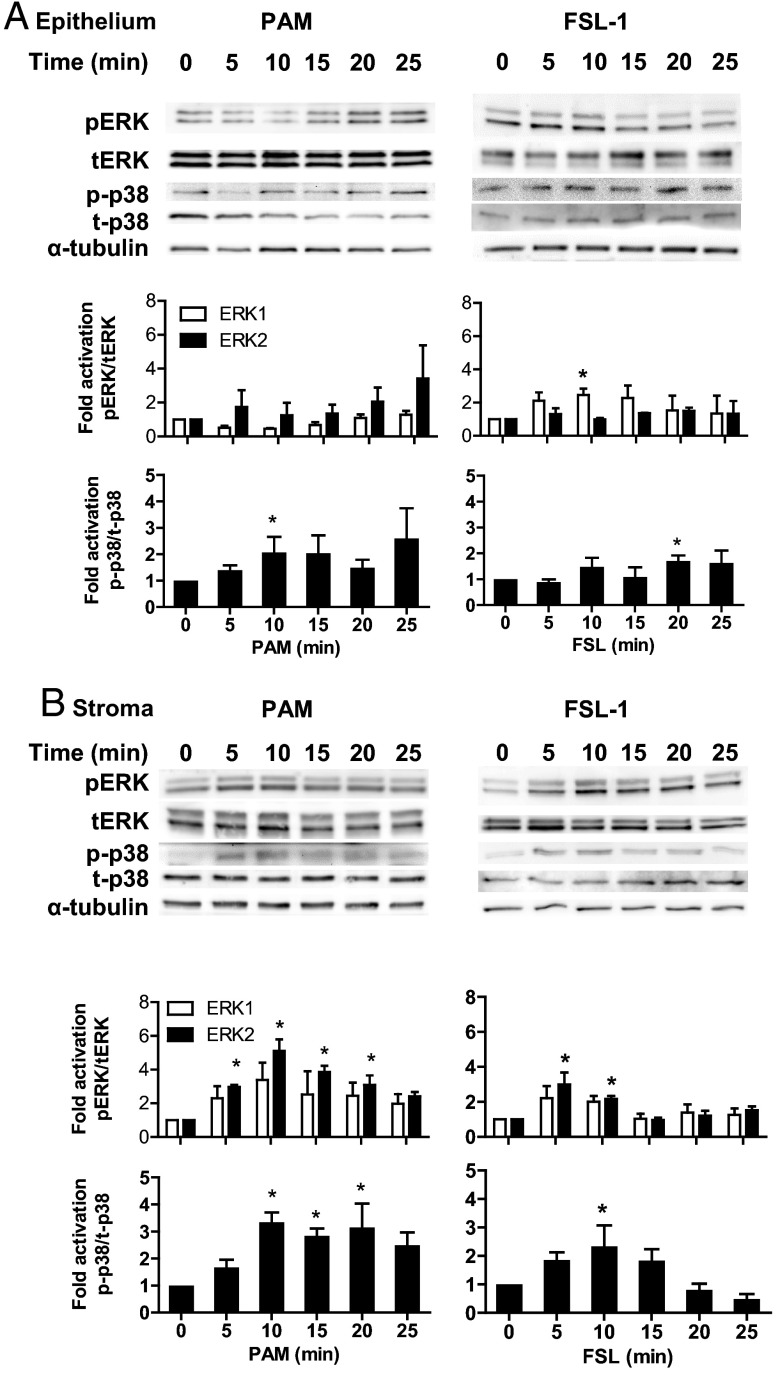
Activation of MAPK in endometrial cells treated with lipopeptide PAMPs. Endometrial epithelial cells (A) and stromal cells (B) were collected 0, 5, 10, 15, 20, or 25 minutes after treatment with 100 ng/mL PAM or 100 ng/mL FSL-1. The protein from the cells was analyzed by SDS-PAGE and immunoblotted with antibodies against total and phosphorylated forms of p38 (t-p38 and p-p38) and ERK1/2 (tERK1/2 and pERK1/2; □, tERK2; ■, pERK2), and α-tubulin as visual confirmation of the precision of protein loading and transfer. The image for each cell type is representative of 3 independent experiments for PAM (left panel) or FSL-1 (right panel), and the histograms represent the mean ± SEM of densitometric analysis of the ratio of phosphorylated p-p38 to t-p38, pERK1 to tERK1 or pERK2 to tERK2, expressed as fold activation compared with time 0. Values differ from time 0 when data were analyzed by ANOVA, using the Dunnett pairwise multiple comparison *t* test: *, *P* < .05.

**Figure 6.**
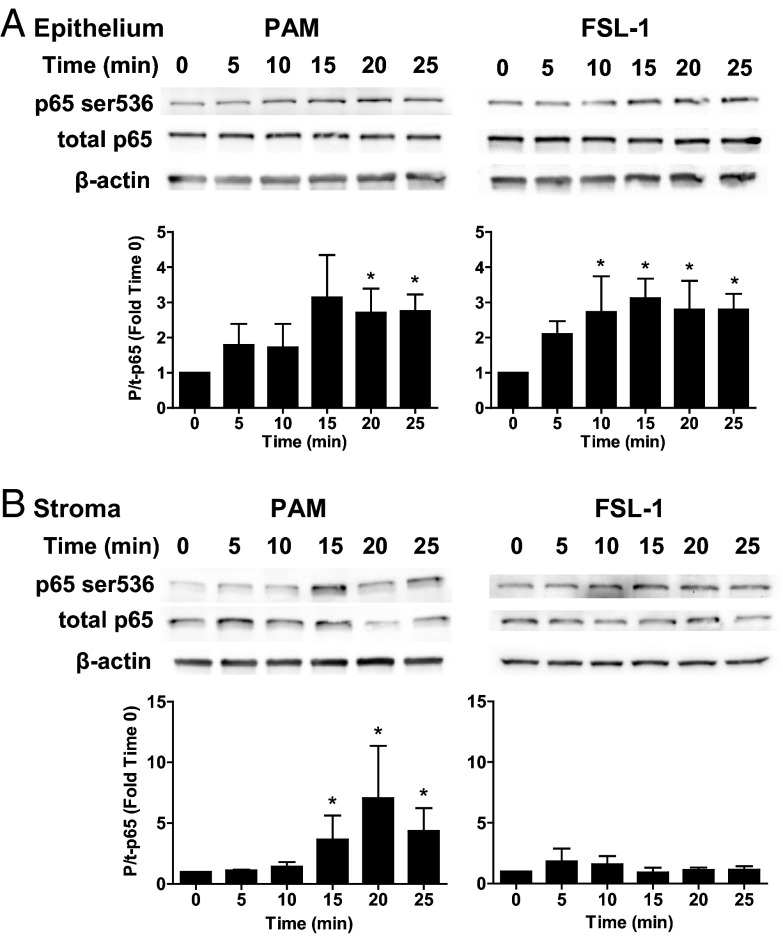
Activation of p65 NFκB in endometrial cells treated with lipopeptide PAMPs. Endometrial epithelial cells (A) and stromal cells (B) were collected 0, 5, 10, 15, 20, or 25 minutes after treatment with 100 ng/mL PAM or 100 ng/mL FSL-1. The protein from the cells was analyzed by SDS-PAGE and immunoblotted with antibodies against total and phosphorylated forms of p65 NFκB (t-p65 and p-p65) and β-actin as visual confirmation of the precision of protein loading. The image for each cell type is representative of 3 independent experiments for PAM (left panel) or FSL-1 (right panel), and the histograms represent the mean + SEM of the densitometric analysis of the ratio of phosphorylated to total p65 NFκB, expressed as fold activation compared with time 0. Values differ from time 0 when data were analyzed by ANOVA, using the Dunnett pairwise multiple comparison *t* test: *, *P* < .05.

Activation of MAPK and NFκB signaling was examined to seek further evidence for functional TLR responses to lipopeptides in endometrial cells. Epithelial cells had more phosphorylated p38 after treatment with PAM and more phosphorylated p38 and phosphorylated ERK1 after treatment with FSL-1 (Figure 5A). Stromal cells had increased abundance of phosphorylated ERK2 and p38 after treatment with PAM or FSL-1 (Figure 5B). Epithelial cells had increased abundance of phosphorylated p65 NFκB after treatment with PAM or FSL-1 (Figure 6A). Stromal cells had increased abundance of phosphorylated p65 NFκB after treatment with PAM but not FSL-1 (Figure 6B).

### MAPK inhibitors limit cellular response to lipopeptides

**Figure 7.**
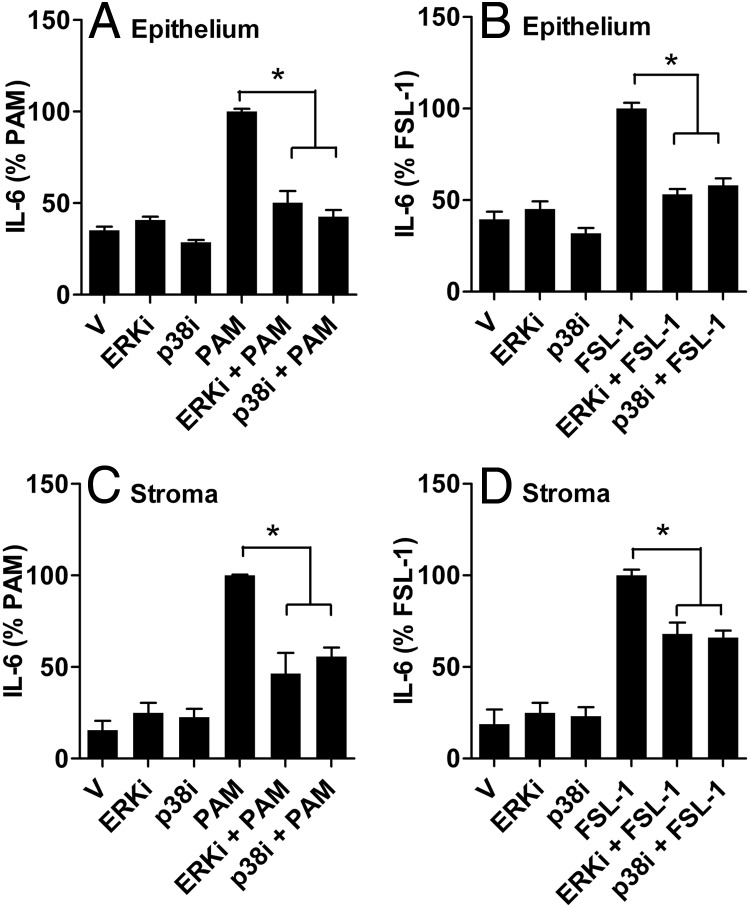
Attenuation of endometrial cell responses to lipopeptides by inhibition of MAPK. Endometrial epithelial (A and B) or stromal cells (C and D) were treated for 30 minutes in medium containing vehicle (V), ERK1/2 inhibitor (ERKi) (ERK activation inhibitor peptide I, 10 μM) or p38 inhibitor (p38i) (InSolution SB 203580, 10 μM) and then were cultured in the same treatment for 6 hours in control medium or medium containing 100 ng/mL PAM (A and C) or 100 ng/mL FSL-1 (B and D). Supernatants were harvested to measure the accumulation of IL-6 by ELISA, and results are expressed as a percentage of treatment with PAM (A and C) or FSL-1 (B and D). Data are presented as mean + SEM percentages and represent 3 independent experiments. Values differ from those for PAMP when data were analyzed by ANOVA using the Dunnett pairwise multiple comparison *t* test: *, *P* < .05.

The accumulation of IL-6 in the supernatant of endometrial stromal cells was used as an exemplar to further evaluate the role of MAPK signaling pathways in the response to lipopeptides. Treatment with PAM or FSL-1 for 6 hours increased the accumulation of IL-6 in supernatants of epithelial cells (control, 27.9 ± 5.3 pg/mL; PAM, 82.6 ± 9.5 pg/mL; and FSL-1, 76.8 ± 9.4 pg/mL; n = 6, *P* < .05) and stromal cells (control, 16.8 ± 3.7 pg/mL; PAM, 503.3 ± 99.1 pg/mL; and FSL-1, 502.3 ± 180.8 pg/mL; n = 6, *P* < .05). Inhibitors targeting ERK1/2 or p38 reduced the accumulation of IL-6 in supernatants of epithelial (Figure 7, A and B) or stromal cells (Figure 7, B and D) treated with PAM or FSL-1. Cell survival was not significantly affected by the inhibitor for ERK1/2 (118.5 ± 11.9% [epithelium MTT assay] and 104.6 ± 6.9% [stroma] of vehicle) or p38 (104.6 ± 13.5% [epithelium] and 114.8 ± 6.0% [stroma] of vehicle), and the inhibitors did not significantly affect cell survival or cell density in culture wells in the presence of PAM or FSL-1 (OD in the range of 94.1 to 121.2% of vehicle for epithelium and 103.4 to 111.0% for stroma).

### Ovarian steroids

**Figure 8.**
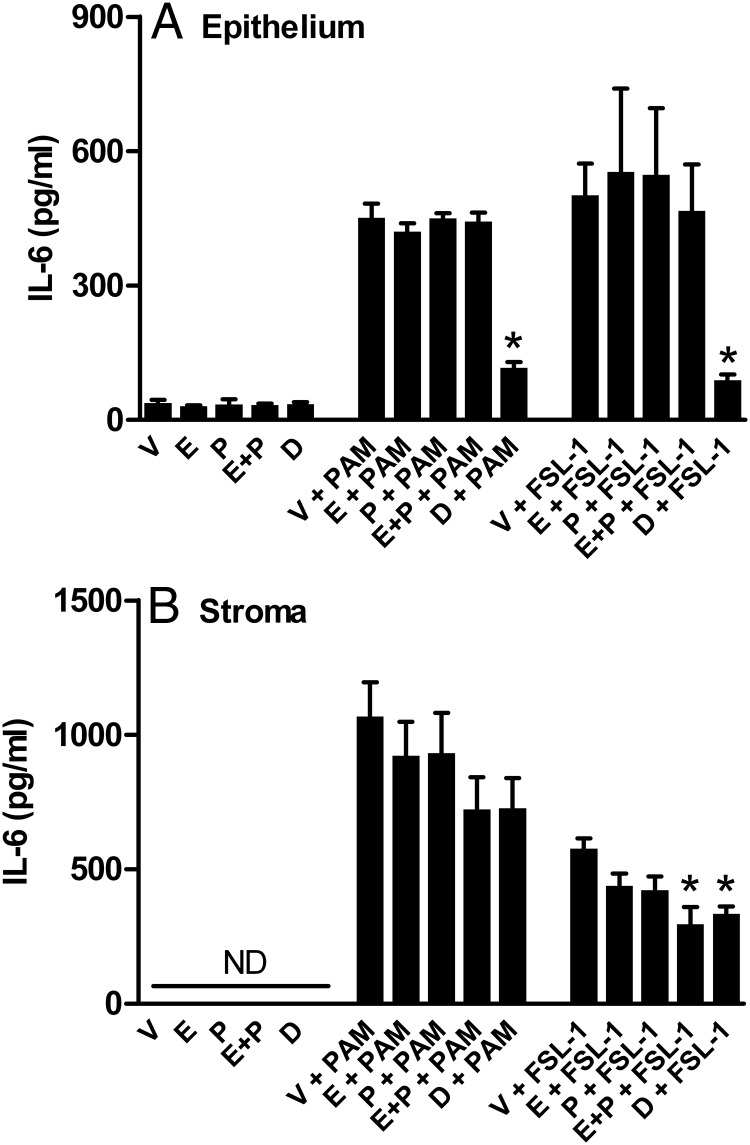
Ovarian steroids and endometrial cell responses to lipopeptides. Endometrial epithelial (A) or stromal cells (B) were treated for 24 hours in medium containing vehicle (V), estradiol (E, 3 pg/mL), progesterone (P, 5 ng/nl), estradiol and progesterone (E + P), or dexamethasone (D) and then were cultured with the same treatment for a further 24 hours in control medium or medium containing 100 ng/mL PAM or 100 ng/mL FSL-1. Supernatants were harvested to measure the accumulation of IL-6 by ELISA, and data are presented as mean + SEM pg/mL and represent 3 independent experiments. Values differ from those for vehicle, within treatment, when data were analyzed by ANOVA using the Dunnett pairwise multiple comparison *t* test: *, *P* < .05.

The stage of the estrous cycle may modulate inflammatory responses in the endometrium (32). Therefore, the impact of estradiol, progesterone, or a combination was examined in cells treated with PAM or FSL-1. As expected, treatment for 24 hours with PAM or FSL-1 increased the accumulation of IL-6 in supernatants of epithelial cells (*P* < .001) (Figure 8A) and stromal cells (*P* < .001) (Figure 8B) compared with control medium. However, the ovarian steroids did not significantly affect the accumulation of IL-6 in epithelial cells, although dexamethasone considerably reduced the response to PAM or FSL-1 (Figure 8A). A combination of estradiol and progesterone modestly reduced the accumulation of IL-6 by stromal cells treated with FSL-1 but not with PAM (Figure 8B).

## Discussion

Endometrial epithelial and stromal cells encounter many species of bacteria that infect the uterus around the time of coitus or after parturition (1, 2, 33). The present study examined whether these epithelial and stromal cells have roles in the detection and response to bacterial lipopeptides. The supernatants of primary endometrial cells accumulated IL-6 and IL-8 in a concentration-dependent manner when treated with triacylated and diacylated lipopeptides. The cellular responses to the triacylated lipopeptide PAM were mediated by TLR1 and TLR2. However, unlike mouse or human cells, the bovine endometrial cell responses to the diacylated lipopeptide FSL-1 involved TLR1, as well as TLR2 and TLR6. The functional responses to the lipopeptides were supported by evidence of activation of intracellular MAPK and NFκB signaling, and inhibitors targeting ERK1/2 or p38 blunted the IL-6 cellular response to lipopeptides. However, ovarian steroids had little effect on the cellular response to lipopeptides, although dexamethasone blunted epithelial cell IL-6 accumulation. These data support the hypothesis that epithelial and stromal cells have a generalized role in innate immunity to initiate inflammatory responses to a wide range of bacteria that infect the endometrium, by sensing bacterial lipopeptides using TLR2 in concert with TLR1 and TLR6.

Gene transcripts for *IL6* and *IL8* are more abundant in the endometrium of cattle with uterine disease than in normal animals (15, 16) and in endometrial explants treated with heat-killed Gram-positive bacteria or bacterial lipopeptides (34). Similarly, in vitro organ culture of bovine endometrium with clinical isolates of Gram-negative or Gram-positive bacteria stimulated accumulation of IL-6 and IL-8 protein (32). Previously we established that endometrial epithelial and stromal cells respond to pathogenic *E. coli* via TLR4-dependent responses to LPS (17, 18, 35). However, it was not clear whether endometrial cells could detect a wider diversity of bacteria, including Gram-positive bacteria and *Mycoplasma*. Lipopeptides constitute 1% to 3% of bacterial genomes with roles in physiology and virulence, with triacylated lipopeptides predominant in Gram-negative species, whereas mainly diacylated forms are found among Gram-positive bacteria and *Mycoplasma* (9). In the present study, triacylated and diacylated lipopeptides stimulated epithelial and stromal cell secretion of IL-6 and IL-8 in a concentration-dependent manner, with a cellular sensitivity similar to the response to the prototypical PAMP, LPS. Although the synthetic lipopeptides are obviously not found in vivo, they mimic the inflammatory responses of cells to native bacterial lipopeptides but avoid multiple issues associated with contamination of native lipopeptide preparations with ligands that bind other TLRs (14). In the present study, the lipopeptide concentrations tested in vitro spanned the range recommended by the manufacturer for working with immune cells. Bacteria are highly abundant in uterine disease, often with >1000 bacterial colonies cultured from a uterine swab, and the LPS concentrations reflected those measured in the uterine lumen of diseased cattle (24). The endometrial cells had little or no hematopoietic cell contamination, as determined by FACS analysis for CD45. The cytokeratin expression showed that the epithelial and stromal cells were distinct, and, as expected, epithelial cells were positive for cytokeratin and vimentin, whereas most stromal cells expressed vimentin (36). The endometrial cell cytokine and chemokine responses to bacterial lipopeptides were typical of cells involved in innate immunity across mammals (5, 6). It is not clear whether endometrial stromal cells from other species respond to lipopeptides, although PAM-stimulated rat endometrial epithelial cells release CCL20 (37). The IL-6 and IL-8 secretion by endometrial cells in response to lipopeptides in vitro, in the present study, mimics the increased expression of *IL6* and *IL8* evident in the endometrium in vivo when the uterus is infected postpartum with a wide range of bacteria that posses lipopeptides. Secretion of IL-8 attracts neutrophils from the peripheral circulation, whereas IL-6 has multiple roles including activation of neutrophils and simulation of the acute-phase response (5, 6, 38). Attraction of neutrophils by IL-8 is thought to form the pus found in the uterus of affected animals, and IL-6 induces the acute-phase response, which is evident in animals with postpartum metritis (1, 4). Postpartum endometritis is often caused by Gram-negative bacteria, and endometrial cells detect and respond to LPS (18). However, *E. coli* are not always isolated from the postpartum uterus, and Gram-positive bacteria also cause uterine disease (4). The ability of endometrial cells to detect and respond to bacterial lipopeptides via TLR2/TLR1 and TLR2/TLR6 is probably important for the onset and persistence of endometritis in cattle.

Triacylated lipopeptides are bound by the extracellular domain of TLR2/TLR1 heterodimers, whereas TLR2 in concert with TLR6 binds diacylated bacterial lipopeptides, at least in mice and humans (10, 11, 39). These germline-encoded, transmembrane receptors on the surface of cells have an extracellular leucine-rich repeat domain typical of most TLRs (5, 6, 10). Crystallization of TLR2/TLR1 and PAM reveals that 2 ester-bound acyl chains of the lipopeptide interact with a hydrophobic pocket within TLR2, whereas the amide-bound acyl chain is inserted into a narrow channel in TLR1 (10). Diacylated lipopeptides interact with the lipid-binding pocket of mouse TLR2 in the same way as PAM, but TLR6 seems unlikely to contain a lipid-binding channel, and so the head group of lipopeptides may play a more important during recognition by TLR2/TLR6 heterodimers. In the present study, siRNA was used to target each TLR, and, as expected from work on mice and human immune cells, TLR2 was important for the endometrial cellular responses to PAM and FSL-1, whereas TLR1 was also involved in the response to PAM. Surprisingly, the cellular responses to FSL-1 involved not only TLR6 but also TLR1. However, the present data are in agreement with a recent report that human embryonic kidney cells stably transfected with bovine TLR2 and TLR1 recognized diacylated and triacylated lipopeptides, which stimulated the production of IL-8 (40). The difference between species probably reflects the diversity of TLRs between cattle and mice or humans (41). However, the concept of variation in ligand specificity is not unique to *B. taurus* because chicken TLR2/TLR1 heterodimers also recognize FSL-1 (41). Of course, the TLRs have been under evolutionary selection pressure since the origin of innate immune receptors in lower organisms. When TLR structures are compared across species, the most divergent part is the extracellular domain involved in ligand binding, whereas the regions involved in heterodimerization and intracellular signaling are conserved. It is thought that the differences in ligand specificity between species are the result of selective pressures for host-specific pathogens, and the less discriminatory sensing by TLR1 may reflect the microbial environment cattle have encountered during evolution (41).

To provide further evidence of the functional role of TLR2 in endometrial cells, the phosphorylation of MAPKs and NFκB was examined (12). Indeed, there was evidence of increased phosphorylation of p38, ERK2, and p65 NFκB in response to the lipopeptides, although there was cell-specific variation. These observations are in accord with similar intracellular signaling responses in murine and human cells. In addition, inhibitors of p38 and ERK1/2 activity reduced the accumulation of IL-6 in response to PAM or FSL-1, without significantly affecting epithelial or stromal cell survival.

Steroids regulate endometrial function during ovarian cycles and pregnancy, and progesterone is thought to dampen the immune response, whereas estrogens are protective against infections (42). Therefore, the lack of effect of steroids on innate immunity in the present study was surprising, particularly because dexamethasone was effective for reducing IL-6 responses to lipopeptides in the epithelial cells. The TLRs are part of an ancient system for protection against microbes and evolved in animals well before viviparity (7). Furthermore, suppression of innate immunity would expose animals to the risk of overwhelming infection (43). Thus, on reflection, steroids might have coevolved with adaptive immunity to modulate inflammation, but innate immunity may be fundamental for survival of animals.

In the present study, the concentrations of TNFα and IL-1β were below the limits of detection of the assay, even for cells treated for 24 hours with either PAMP. The lack of protein accumulation probably reflects the rather brief increase in *TNF* mRNA expression, even when endometrial cells are treated with LPS (44). Indeed, the association between *TNF* mRNA expression and disease is inconsistent in endometrial biopsies (15, 45). The lack of IL-1β protein was not surprising because secretion of mature IL-1β is dependent on caspase-1 cleavage of pro-IL-1β after formation of the intracellular inflammasome complex, which requires a second stimulus such as cell damage as well as the presence of PAMPs (46). In the present study, there was no evidence of an effect of the PAMPs on endometrial cell survival, although in some cell lines bacterial lipopeptides are associated with increased apoptosis (12). Because the endometrium is undergoing considerable remodeling during estrous cycles or the postpartum period, future studies could explore whether PAMPs affect cell survival and extracellular matrix remodeling.

In conclusion, endometrial epithelial and stromal cells mounted cellular responses to bacterial lipopeptides typical of innate immunity with the accumulation of IL-6 and IL-8. The cellular responses to the lipopeptides involved TLR2 in concert with TLR1 and TLR6 and with more plasticity in the TLR-dependent recognition of FSL-1 for bovine cells than anticipated from work with mice or humans. The bacterial lipopeptides activated MAPK and NFκB intracellular signaling pathways in endometrial cells, and inhibitors targeting ERK1/2 or p38 limited the inflammatory responses. Bacterial lipopeptides are important for innate immunity because they are produced by all bacteria, and thus TLR2 has generalized roles in detecting bacteria, usually involving monocytic immune cells (12). The findings in the present study that endometrial epithelial and stromal cells sense and respond to bacterial lipopeptides now provide a mechanistic explanation linking endometritis with infections by Gram-positive as well as Gram-negative bacteria.
